# Goal-directed learning and obsessive–compulsive disorder

**DOI:** 10.1098/rstb.2013.0475

**Published:** 2014-11-05

**Authors:** Claire M. Gillan, Trevor W. Robbins

**Affiliations:** 1 Department of Psychology, New York University, 6 Washington Place, New York, NY 10003, USA; 2 Behavioural and Clinical Neuroscience Institute, University of Cambridge, Downing Street, Cambridge CB2 3EB, UK; 3 Department of Psychology, University of Cambridge, Downing Street, Cambridge CB2 3EB, UK

**Keywords:** habit, obsessive–compulsive disorder, goal directed, compulsivity

## Abstract

Obsessive–compulsive disorder (OCD) has become a paradigmatic case of goal-directed dysfunction in psychiatry. In this article, we review the neurobiological evidence, historical and recent, that originally led to this supposition and continues to support a habit hypothesis of OCD. We will then discuss a number of recent studies that have directly tested this hypothesis, using behavioural experiments in patient populations. Based on this research evidence, which suggests that rather than goal-directed avoidance behaviours, compulsions in OCD may derive from manifestations of excessive habit formation, we present the details of a novel account of the functional relationship between these habits and the full symptom profile of the disorder. Borrowing from a cognitive dissonance framework, we propose that the irrational threat beliefs (obsessions) characteristic of OCD may be a consequence, rather than an instigator, of compulsive behaviour in these patients. This lays the foundation for a potential shift in both clinical and neuropsychological conceptualization of OCD and related disorders. This model may also prove relevant to other putative disorders of compulsivity, such as substance dependence, where the experience of ‘wanting’ drugs may be better understood as post hoc rationalizations of otherwise goal-insensitive, stimulus-driven behaviour.

## 1. Introduction

‘Compulsivity’, although a young concept, has captured the imagination of researchers fast in the fields of psychology, psychiatry and neuroscience, and this burgeoning interest is reflected in the dramatic rise in the number of articles referring to this term in the last 5 years in particular. A range of maladaptive human behaviours are now popularly considered to be examples of compulsivity, including psychiatric problems such as drug use in substance-dependent individuals [1], repetitive behaviours [2] and tics [3] in Tourette's syndrome, excessive and restrictive eating behaviours in eating disorders [4] and the repetitive, avoidance behaviour seen in the eponymous disorder of compulsivity, obsessive–compulsive disorder (OCD) [5]. Although the list of overt behaviours that have been classified as compulsive is quite varied, there is consensus that compulsivity is ‘a hypothetical trait in which actions are persistently repeated despite adverse consequences’ [6]. In OCD, hand-washing behaviour, for example, is often continued in spite of the development of abrasions to the skin, along with the more typical loss of occupational and social function that is associated with the time taken to perform most compulsions. Although compulsivity has been successfully operationalized in terms of the resistance of this kind of behaviour to punishment, there is disagreement in the literature regarding the psychological mechanism which gives rise to compulsive behaviour. In this article, we aim to address this issue with reference to OCD, via recent research aimed at elucidating the neural and psychological basis of compulsive behaviour. While the majority of data discussed will pertain to OCD, the issues raised may also be relevant for understanding other purported disorders of compulsivity, for which empirical data in patient populations are currently lacking.

There are arguably two main schools of thought regarding the underlying mechanism that leads to compulsive behaviour. The first school is ‘cognitive’ and purports that compulsivity is mediated by dysfunction in the assignment of value to available alternatives, that is, the compulsive individual may view the cost of cessation of behaviour to be higher than the benefits thereof. In this sense, the choice to continue the behaviour is purposeful and goal directed; it is simply deemed compulsive by the outside observer (i.e. a clinician or society) to whom this choice appears suboptimal. Concordant with this view, many researchers have speculated that compulsions in OCD are performed to reduce the likelihood that an unwanted, or feared, consequence will take place [7–9]. Disordered valuation or ‘cognitive bias’ is primary and motivates patients to perform avoidance compulsions as a form of coping with these distressing thoughts [10]. ‘Cognitive bias’ is a broad term; it may subsume an exaggerated sense of personal responsibility [10] over the environment and thought–action fusion, the belief that thinking about something is equivalent to doing it [11,12]. Cognitive bias accounts of OCD purport that patients with OCD initially experience obsessions associated with potential threat or discomfort, and as a consequence of such distressing beliefs, anxiety is engendered. As a consequence of the two factor theory of avoidance [10], which suggests that Pavlovian fear arises first and motivates avoidance in healthy animals, compulsions are carried out as goal-directed, purposeful, attempts to reduce the likelihood of threat, or more generally to provide relief [13].

This article will argue that contrary to the cognitive account, OCD does not necessarily arise from faulty value attribution, or ‘cognitive bias’, but rather, it may result from goal-directed dysfunction that interacts with anxiety and irrational belief in a manner not hitherto discussed in the literature. This position holds that patients with OCD largely understand the relative value of the available outcomes and the cost of actions, and aim to promote expected values of outcomes and desist from compulsive behaviour, but cannot exert the necessary control over their actions to realize this goal. One way in which this lack of control over behaviour might arise is as a result of an imbalance between two fundamental associative learning systems that are relatively well characterized in both psychological and neurobiological terms.

## 2. Neurobiological parallels: habit and obsessive–compulsive disorder

Habits are responses that are automatically triggered by stimuli and are considered the functional reciprocal of goal-directed behaviours that are intentional, considered, and as the name suggests, sensitive to the value of prospective goals [14]. The relative control that these two systems exert over behaviour have been described using a number of different theoretical frameworks [15–18]. However, in spite of differences in terminology, these frameworks converge around a common theme; that animals use both *reflective* and *reflexive* modes of action selection [19,20]. Goal-directed behaviour is more accurate, but that accuracy requires effort and attention. It follows that this mode of action selection suffers in times of stress [21], perhaps as a result of increases in working memory load [22], and is seen later in childhood development than habit learning [23]. Most famously, however, goal-directed control over action subsides as we become comfortable with repetitive action following over-training of the stimulus–response pair [24], and when outcomes are less tightly coupled to responses [25].

**Figure 1.**
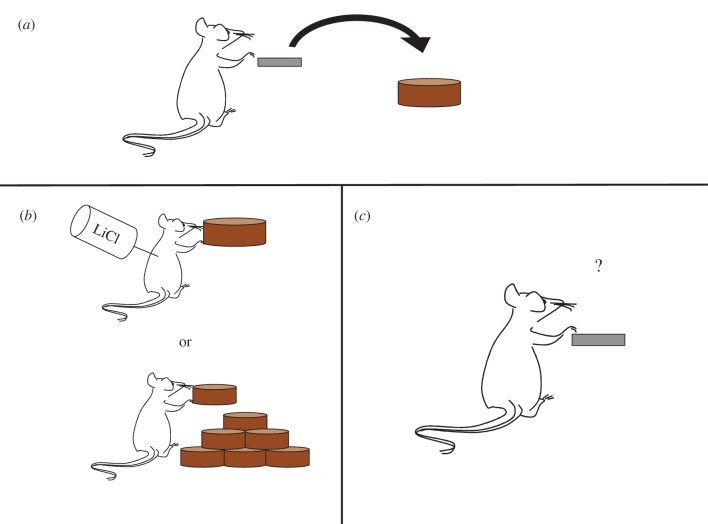
Outcome-devaluation procedure. (*a*) Animals are trained to press a level to gain food pellets. (*b*) Food pellets are typically devalued by, for example, pairing with lithium chloride, which induces nausea, or by feeding to satiety. (*c*) Animals are tested on extinction. Continued responding reflects insensitivity to outcome devaluation, and thus dominant control of the habit system.

In 2000, Graybiel & Rauch [26] proposed the theory that OCD can be characterized as a disorder of maladaptive habit learning on the basis of neurobiological parallels between the brain regions implicated in OCD and the then proposed functional loci of repetitive behavioural habits, namely regions comprising the ‘fronto-striatal circuits’ [27]. Since then, a plethora of studies in rodents and humans has been conducted to elucidate the neural basis of habit formation and has reached consensus that a shift from associative to sensorimotor fronto-striatal circuits mediates the transition from goal-directed to habitual control over behaviour [28,29]. To test for habits, a procedure called outcome devaluation is most commonly used (another is that of contingency degradation [30]; figure 1).

In this procedure, the outcome of a given action is made undesirable and continued responding for the stimulus is measured in an extinction test. When behaviour is under the control of the goal-directed (action–outcome) system, responding to stimuli that produce devalued outcomes should decline, whereas if habits (stimulus–response) are dominant, behaviour will become insensitive to the value of the outcome [31]. Research in rodents using this technique has revealed an important double dissociation between medial and lateral subregions of the dorsal striatum in the balance between goal-directed and habitual control over behaviour. Specifically, habitual responding can be induced in rodents by lesions of the dorsomedial striatum (DMS: caudate in primates) [32], suggesting that this region is critical for goal-directed action control. Disrupting activity in the dorsolateral striatum (DLS: putamen in primates), on the other hand, preserves sensitivity to outcome value in rodents, even after extended training [33,34]. In the rodent prefrontal cortex, although the literature is less clear, there has been some suggestion of a similar double dissociation between the prelimbic and infralimbic cortices, with the former being associated with goal-directed learning (but see [35]) and the latter necessary for the execution of habits [36–38]. However, these data are contentious and it is presently unclear if and how rodent–primate homologies in the prefrontal cortex can be legitimately drawn, and so these data are difficult to interpret. The prelimbic cortex has been suggested to correspond to the human area 32 (a portion of anterior cingulate cortex) and the infralimic (IL) cortex to primate area 25 (although there is some doubt also about this). Rather than processing stimulus–response associations directly, some have proposed that the IL may arbitrate between controllers, inhibiting the goal-directed system in favour of performing previously reinforced actions [37,39]. On the other hand, the IL is also implicated in both fear and appetitive (cocaine) extinction [40], and this role is entirely consistent with an inhibitory role for IL in the learning and expression of stimulus–outcome and action–outcome associations. In mice, recent data have highlighted an important role for the medial orbitofrontal cortex (OFC) (more rostral to the IL cortex in the rat) whereby inhibition of activity in this region interferes with goal-directed behavioural control, and furthermore, neuronal firing rates in the DMS, DLS and OFC in healthy animals dynamically change in concert with a shift between goal-directed and habitual actions in a manner suggestive of a key role for the OFC in goal-directed action selection [41]. In line with this finding, in the rhesus monkey, lesions to areas 11 and 13 of the OFC cause impairments in goal devaluation, whereas lesions of area 14 disrupt extinction [42].

Studies using functional magnetic resonance imaging (fMRI) in humans are broadly consistent and suggest a key role for subregions of the ventromedial prefrontal cortex (vmPFC) in goal-directed control over action. Using selective satiety to devalue outcomes, Valentin *et al*. [43] showed that activity in portions of the medial, central and lateral OFC were sensitive to the choice of actions that led to valued or devalued outcomes, suggesting these regions play a key role in determining the incentive value of outcomes in goal-directed decision-making. Another study used incongruent associations to create conflict in the goal-directed system, forcing subjects to rely instead on habits. Brain activation was compared for activation during these trials to congruent trials, devoid of conflict. The authors found that a more posterior portion of the vmPFC, corresponding to the perigenual anterior cingulate was more active when participants were carrying out goal-directed as opposed to habitual actions [44]. Other studies using computational analysis have similarly revealed that the anterior caudate nucleus, in addition to the medial OFC, and more rostrally, the medial prefrontal cortex, track the level of contingency between actions and outcomes [45–47]. Few studies, however, have been able to elucidate the neural basis of habits in humans, save for reductions in activation in goal-directed subregions of the vmPFC and caudate described above. One study, however, compared groups who received brief (1 day) versus extended (3 days) training on a free-operant habit task for food outcomes. In a subsequent devaluation test, Tricomi *et al*. [48] observed that activity in the putamen increased when behaviour became autonomous from outcome value following over-training, a finding mirrored in the lack of behavioural sensitivity in the over-trained group. More recently, de Wit *et al*. [49] found convergent evidence observing that increased white matter tract strength between the putamen and premotor regions was predictive of habitual control over behaviour in healthy humans, while the strength of connectivity between the medial prefrontal cortex and caudate predicted goal-directed behavioural choice. To summarize, there is good cross-species consistency regarding the importance of the putamen for stimulus–response habit learning and the caudate for goal-directed action selection. The role of the prefrontal cortex is less clear (and less well specified), but in humans the data converge on an important role for the vmPFC in goal-directed control over behaviour, particularly the medial portions of the OFC and prefrontal cortex.

The dominant neuroanatomical model of OCD centres on, but is not limited to, abnormalities within the regions involved in the balance between goal-directed behaviour and habits. Broadly speaking, neurobiological changes associated with OCD have been identified within circuits that run from the frontal lobes to the striatum, and via direct and indirect pathways to the thalamus and back to the frontal cortex, the ‘fronto-striatal loops’ [27,50,51]. In terms of specific loci, the caudate nucleus and the orbital gyrus are the most consistent regions where OCD patients show abnormal patterns of functional activation [52]. Evidence for this comes primarily from symptom provocation studies [53–56] and treatment response studies following pharmacotherapy and behavioural therapy [57–59] using positron emission tomography and single photon emission computed tomography. In terms of structural brain changes, a recent meta-analysis of voxel-based morphometry studies revealed that OCD patients have increased grey matter volumes in the putamen (extending to the caudate nuclei) relative to healthy controls and other anxiety disorder groups [60], whereas all anxiety groups (including OCD) showed common decreases in dorsolateral prefrontal and anterior cingulate cortex. Another meta-analysis found increased volume of the OFC, putamen and insula in OCD patients, which was a function of age, such that the normal loss of volume was not observed in patients as they aged. This suggests the possibility that these changes may reflect altered neuroplasticity associated with disease duration, for example the performance of compulsive behaviours throughout the lifespan [61]. We will not summarize the findings of fMRI studies in OCD here because no studies to date have investigated the neural correlates of habit learning in this patient group. However, we will describe some studies that may be tangentially relevant later in this review.

## 3. Experimental evidence for goal-directed dysfunction in obsessive–compulsive disorder

**Figure 2.**
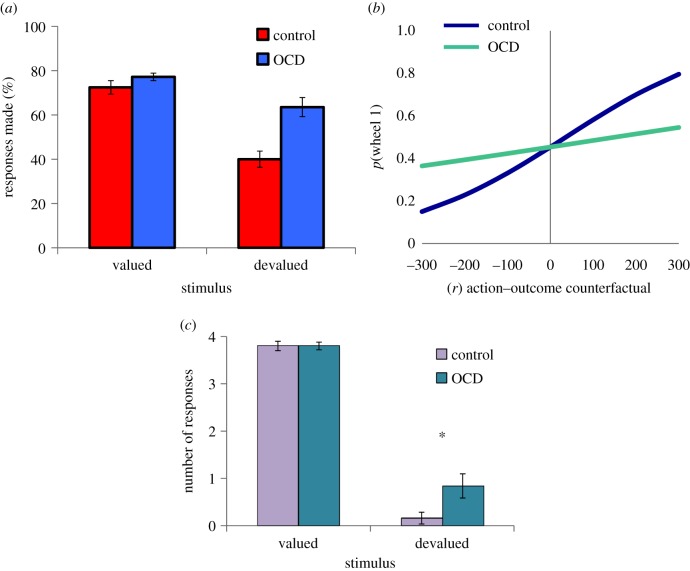
Experimental tests of goal-directed dysfunction in OCD. Reproduced with permission from [48–50]. (*a*) OCD patients show a tendency towards habit formation following appetitive instrumental training. Habits are assessed using an outcome-devaluation test wherein one outcome is ‘devalued’ and another remains valuable. Responding to stimuli that predict devalued outcomes is evidence for dominant stimulus–response habit associations [62]. (*b*) In an economic choice task, OCD patients exhibit impaired use of prospective action–outcome comparisons relative to controls [63]. (*c*) OCD patients exhibit dominant stimulus–response habit associations in a devaluation test following instrumental shock avoidance training [64]. Error bars denote standard error of the mean.

The first study to directly test the contribution of the goal-directed system to OCD was carried out using an appetitive instrumental learning task [62], which has been since shown to rely upon white matter tract connectivity within and between the fronto-striatal circuits [49]. Following trial and error learning of positively reinforced stimulus–response–outcome associations, subjects were given a series of tests to determine the contribution of goal-directed (action–outcome) and habitual (stimulus–response) associations to instrumental learning. The results indicated that OCD patients have a significant bias towards stimulus–response learning, at the expense of acquiring action–outcome associations. This result was evident across three independent tests. In an outcome-devaluation (‘slips-of-action’) test, OCD patients had deficits in their ability to refrain from responding to outcomes that were no longer worth any points (i.e. devalued; figure 2*a*). This result was compounded by behavioural and explicit tests of contingency knowledge, where OCD patients' action–outcome associative knowledge was impaired, but their stimulus–response knowledge was intact.

Further support for the notion that a goal-directed disturbance plays a role in OCD was provided in a follow-up study which assessed counterfactual decision-making in OCD patients on an economic choice paradigm [63,65]. The design of the paradigm was such that there was no repetition of stimulus–response–outcome pairings, but rather, participants had to choose between two wheels depicting points and their respective probabilities. In this way, we could assess goal-directed choice behaviour in the absence of the potentially confounding influence of concurrent formation of habits over the course of training. Rather than using outcome devaluation, this study adopted a computational approach to understanding goal-directed behaviour, wherein trial-by-trial choice behaviour was used as the dependent measure. Goal-directed behaviour was operationalized as the degree to which ‘potential regret’ influences choice behaviour. Potential regret is a goal-directed computation that relies upon the comparison of prospective action–outcome states, that is, the ability to simulate and compare options in a decision tree. Healthy adults are known to use this computation to reduce their chances of experiencing regret in this decision environment [63,65]. Consistent with the suggestion that OCD patients have a deficit in goal-directed control over action, the influence of this computation on decision-making was attenuated in OCD patients (figure 2*b*). Rather than being linked to a muted emotional experience of regret, as has been observed in patients with lesions to the OFC who also show this kind of choice behaviour [66], OCD patients experienced regretful trials as being even more aversive than healthy comparison subjects. OCD patients and comparison subjects did not differ in the extent to which their choices were based on the expected value of the available options, suggesting that basic decision processes were not affected in these patients.

A third study tested whether the deficits in appetitive goal-directed behaviour observed in OCD would be evident in avoidance. This question is not trivial because OCD is a disorder of compulsive avoidance rather than reward-seeking behaviour, and habits in avoidance had previously not been experimentally demonstrated in humans or animals. In this paradigm, OCD patients and healthy controls were trained to avoid aversive electrical shocks to their wrists by performing the correct response to a predictive stimulus, constituting negatively reinforced stimulus–response–outcome discriminations [64]. Following an over-training period, participants were tested for habit formation using a selective outcome-devaluation procedure, wherein the shock electrodes were removed from one of their wrists (devalued), but remained connected on the contralateral side (valued). While OCD patients and controls did not differ in the number of avoidance responses made in response to the stimulus that predicted the valued shock, OCD patients made significantly more responses to the stimulus that predicted the devalued shock (figure 2*c*). A devaluation sensitivity test revealed that OCD patients were proficient in their goal-directed control over their responses prior to over-training and that their behaviour became excessively habit-based over the course of over-training. There was no evidence to suggest that habits were driven by associated failures in fear extinction in OCD patients using both physiological (i.e. skin conductance response) and evaluative conditioning (i.e. shock expectancy) measures. However, it is possible that avoidance habits are associated with impaired instrumental extinction in a more general sense, a proposition, which to our knowledge has not yet been tested. It is however unclear if and how these might be parsed experimentally.

Using different methodologies, these three studies show that in OCD there is a consistent shift in balance away from goal-directed associative control over action towards stimulus–response habits. There are a number of potential causes for this imbalance, three of which we will consider now. The first is that OCD patients may have a deficit in action–outcome associative learning, which causes them to rely excessively on stimulus–response links that were previously reinforced. There is ample evidence in support of this possibility, given that explicit knowledge of action–outcome associations is deficient in OCD following instrumental learning, and, furthermore, that these explicit learning deficits were correlated with patients' subsequent failure to show sensitivity to devaluation [62]. Next, an alternative possibility is that excessive stimulus–response learning in OCD might cause patients to lose their sensitivity to action–outcome links, producing deficits in explicit action–outcome knowledge. However, the observation that OCD patients exhibit goal-directed deficits on a decision-making paradigm that does not permit stimulus–response habit learning does not sit well with this interpretation [63]. Rather, these data suggest that a fundamental problem in action–outcome associative learning and/or execution exists in OCD, and it is not dependent on excessive habit formation in the disorder. In line with this account, recent fMRI work has found that OCD patients show under-activation of the ventral striatum during a reward anticipation task that requires goal-directed behaviour [67], and this is remediated by deep-brain stimulation of this region [68]. Conversely, in the avoidance habit experiment described above, habit biases were observed despite intact action–outcome knowledge in patients tested in this study, using a comparatively less complex paradigm compared with the original study on appetitive habit learning. This indicates that habit biases in OCD are not necessarily driven by deficits in goal-directed contingency knowledge. It is plausible then that both habit-based and goal-directed learning may be affected in OCD, however, until a behavioural definition of habit learning that does not rely on an absence of goal-directed control can be formalized, there is perhaps little use in making a distinction between processes that are somewhat reciprocal. The recent ‘model-based, model-free’ reinforcement-learning schema [16], wherein model-based behaviour is hypothesized to map onto goal-directed action and model-free supports habit learning, does not consider the two systems reciprocal, and can therefore assess their independent contributions to choice behaviour. Using this paradigm, there is recent evidence to suggest that model-based control over action is selectively diminished in OCD patients [69]. Although this is a promising avenue for future research, more work is needed to assess how this schema maps onto the habit-based, goal-directed dichotomy, as a direct comparison is currently lacking.

**Figure 3.**
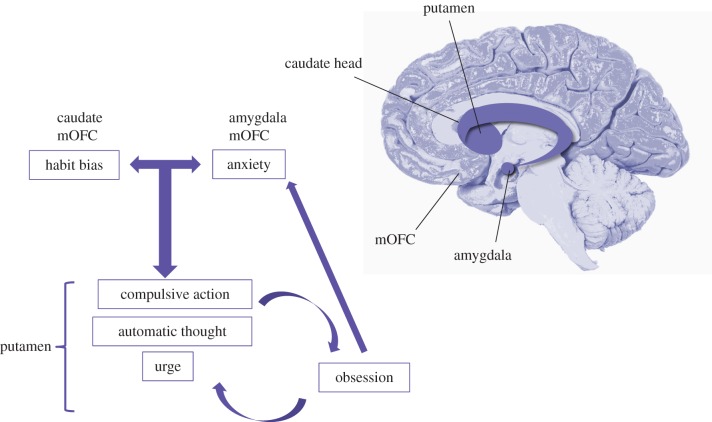
Proposed model of the relationship between core quantifiable traits and symptomatology in OCD. Biases towards habit formation and trait anxiety (although may not be necessary for diagnosis) act in concert to foster compulsive urges, probably supported by the putamen, where action control is transferred from a misfiring (putatively hyperactive) caudate and OFC. Obsessions may be a cognitive interpretation of compulsive urge, which ultimately interacts with anxiety and reinforces the desire to perform compulsions through cognitive dissonance. Brain schematic illustrates important nodes in the fronto-striatal circuits implicated in OCD, of which the mOFC and the caudate are most consistently implicated. Disruption in these regions may be necessary and sufficient for OCD diagnosis, but probably contribute to a range of disorders along the anxious and compulsive spectrums.

A third possibility, which we will touch on only briefly, is that rather than abnormalities in goal-directed control or habits, in OCD the problem could lie in the arbitration between these controllers. Recent evidence suggests that this arbitration is carried out in the frontal polar and inferior lateral prefrontal cortex, which track the reliability of the predictions of model-based and model-free controllers, respectively, and use these signals to inhibit the model-free system, where appropriate [70]. This postulate awaits testing in OCD patients, however, given the neurobiological evidence outlined earlier, it seems more plausible that dysfunctional goal-directed learning processes (outcome valuation, contingency) associated with the caudate and medial OFC are responsible for biases towards habitual responding in OCD (figure 3).

## 4. Habits and compulsive-obsessive disorder ‘COD’

We have previously suggested an alternative way of conceptualizing the functional relationship between obsessions and compulsions in OCD, a model captured by a rearrangement of the letters OCD to COD [6]. This simple reshuffle belies what is a possibly crucial and maybe overdue shift in thinking away from the previously prescribed unidirectional nature of reinforcement that exists in the cycle of OCD symptomatology (figure 3). As outlined in the Introduction, classic cognitive models of OCD posit that obsessions precede compulsions, which are considered active attempts to gain relief from obsessive thoughts. Indeed, it is impossible to ignore the tight coupling between the content of obsessions and compulsions in OCD, which leads to the intuitive inference: ‘I fear contamination and therefore I feel compelled to clean excessively’. Based on recent observations, we propose that the reverse—‘I feel compelled to clean excessively and therefore I *must be* afraid of contamination’—may better capture the OCD phenomenon.

There are two main problems with the current OCD framework ascribed by cognitive models of the disorder, which have led us to consider this alternative possibility. The first is that existing cognitive models rely on the supposition that obsessions drive OCD and compulsions are secondary phenomena [7–9]. But in the three studies of habit formation described above, there is clear evidence that excessive compulsive-like, automatic behaviours develop in OCD patients in the absence of any prior obsessions relating to the experimental task procedures. First, this demonstrates that there is a purely behavioural disturbance in OCD that is independent of obsessionality. Second, OCD is an ego-dystonic disorder; the thoughts experienced and actions performed by patients are discordant with their concept of self, either categorically or proportionally. In other words, patients have insight (although it can be diminished in some cases) into the irrationality of their compulsive actions; they want to stop but cannot exert control over the urge to act. Cognitive models of OCD cannot account for this insight, how a patient can be aware that there lacks a contingency between flicking a light-switch and averting a traffic accident, and yet feel compelled to perform the action.

One way to reconcile this apparent paradox in OCD is to embrace it. Rather than a problematic footnote in the diagnostic criteria of OCD, the ego-dystonic nature of obsessions and compulsions in OCD may more accurately be considered a cardinal feature of the disorder. By definition, habits are behaviours insensitive to contingency and outcome value; in other words, they are ego-dystonic, purposeless acts. We propose that the excessive habit learning reliably observed in these patients captures the divergence between will and action that typifies OCD. We hypothesize that this behavioural disturbance is the critical component of the OCD diagnosis and has its neurobiological basis in the circuits running between the OFC and the caudate, whose (putative) hyper-activation disrupt normal goal-directed behaviour, fostering reliance on habits (figure 3). We will now outline a model of OCD in terms of ‘COD’, in which a tendency towards compulsive habit learning is central and furthermore, we will suggest a mechanism through which the other critical features of the disorder, anxiety and obsessions, can be explained as both propagators and consequences of compulsivity.

Anecdotally, habits are considered to be automatic errors that go below the level of conscious awareness. We make *slips* when we are distracted and may, for example, take a familiar turn in the road instead of driving straight on to our intended destination. Recent data, however, have challenged this popular conception of habits as mere action-slips, finding that habits are associated with a hitherto unreported premonitory ‘urge to respond’ [64]. This urge may be critical in elaborating a model of how compulsive behaviour could give rise to obsessional thinking. To do this, we borrow from cognitive dissonance theory. Cognitive dissonance describes a state of conflict that arises when two or more competing beliefs are simultaneously held, and cognitive dissonance theory states that humans are motivated to reduce this conflict by altering one of these beliefs [71]. This effect is also observed in humans when behaviour contradicts belief. In a forced compliance study, subjects completed an exceptionally monotonous experiment, after which a subset were instructed to tell new participants who were arriving for the study that it was in fact ‘a very interesting and enjoyable experience’. They found that those subjects who were induced to give a positive report to new participants gave more favourable ratings of their own experience in the study on a subsequent questionnaire [72]. This study convincingly demonstrates that in situations of cognitive dissonance, when behaviour contradicts belief, humans alter their beliefs to match their behaviour. As has been elegantly put by others, ‘actions create—not just reveal—preferences’ [73].

The suggestion that cognitive dissonance may arise as a result of compulsivity is not altogether new, but has been previously alluded to in the context of substance dependence. Everitt & Robbins [74] suggested that the subjective urge to consume drugs (i.e. wanting), often considered a precursor to consumption, may be a post hoc rationalization of the objectively ‘out of control’ behaviour. In the case of OCD, the argument is analogous, wherein the irrational thoughts often considered to induce compulsive responding, may in fact be the product of the mind's attempt to resolve the discrepancy between patients' cognitions and their otherwise inexplicable urge to perform compulsive behaviours. Specifically, the experience of the irresistible urge to perform, or the very performance of, compulsive avoidance behaviours may engender cognitive dissonance that is reconciled by the development of a new irrational belief about threat in the environment. This new ‘fear’ makes sense of the need to compulsively perform avoidance responses and may of course contribute to the motivation of subsequent avoidance responding, forming a vicious cycle of sorts.

The avoidance habit paradigm discussed earlier in this article is the only study, to our knowledge, which begins to test this possibility directly [64]. OCD patients and healthy controls were asked to provide an explanation for why they continued to make avoidance responses to the stimulus that predicted the now devalued outcome. While many patients used the word ‘habit’ or variants of it (i.e. automaticity) to describe their actions, a subset reported irrational hypotheses regarding the task procedure. For example, some patients reported that they thought they could still be shocked, in spite of the fact that they had been disconnected from the offending stimulator. These comments become particularly illuminating when considered alongside the ratings of shock expectancy and explicit contingency knowledge tests, which were taken moments before this qualitative question. Patients were unimpaired on these tests, reporting very low likelihood that they could still be shocked following devaluation and having intact knowledge of the contingency structure of the task. Like obsessions in OCD, these irrational threat beliefs were discordant with subjects' knowledge of the task structure. Although these data were collected in an exploratory manner, they provide good early evidence in support of the suggestion that irrational thinking may sometimes be a consequence of habit formation.

It may be surprising to some how irrational threat beliefs such as these could survive without being promptly disconfirmed through experience. However, the fact that compulsions in OCD are avoidant, rather than appetitive, can readily account for this. It is a feature of avoidance that performance of this response naturally precludes the extinction of irrational beliefs about contingency (e.g. fear), because when avoidance responses are continually, but unnecessarily, performed, the only demonstrable contingency the individual is exposed to is one where a state of safety follows the performance of an avoidance response. This prevents exposure to the crucial disconfirming case or extinction, i.e. when a state of safety is also followed by no response [75]. In this way, the maladaptive cycle of compulsions, obsessions and anxiety may be cyclically maintained and reinforced, allowing for irrational fears to propagate and develop into more persistent obsessions over time.

It is possible that anxiety may arise in some patients with OCD as a product of the aforementioned ‘COD’-cycle, induced as a result of, or in tandem with, irrational threat beliefs. However, it is likely that anxiety plays a much more crucial role in OCD than an epiphenomenal one, given the high rate of co-morbidity and common shared heritability that exists between OCD and other anxiety disorders [76] and the elevated level of trait anxiety reliably documented in the disorder itself in the absence of anxiety disorder co-morbidity (e.g. [64]). It has been consistently demonstrated in both humans and other animals that laboratory stressors promote habit formation in healthy subjects [21,77,78]. Although no study to our knowledge has directly tested the contribution of *anxiety* to habit formation, there is evidence to suggest that anxiety biases attention to stimuli, and away from outcomes, potentially causing a similar failure to execute goal-directed behaviours as seen during stress manipulations [79]. Indeed, individuals with anxiety disorders [80], and healthy students with high levels of trait anxiety [81], have deficits in their ability to ignore distracting stimuli, an attentional function, which, like goal-directed behaviour, relies on activation in the prefrontal cortex [81,82]. However, unlike other anxiety disorders, attentional bias has not been consistently observed in OCD [83], suggesting that while anxiety-related mechanisms may contribute to habit biases in some patients, it is unlikely to fully account for them. Indeed, the available empirical data relevant to this issue suggest that there is no direct association between trait anxiety or physiological measures of conditioned fear learning and extinction and habit formation biases in OCD patients [64]. However, as the design of this study was not capable of parsing out Pavlovian (i.e. stimulus–outcome) physiological fear responses from those that are confounded with the instrumental avoidance response, these data are not definitive.

In studies that have examined purely Pavlovian fear conditioning in OCD, the results are not altogether clear. Some studies find that OCD patients have elevated blood oxygen level dependent responses in the amygdala, a limbic structure associated with emotion processing and fear, when viewing disorder-specific stimuli (i.e. symptom–provocation) [84,85]. There is evidence to suggest that amygdala conditioning to more general emotionally relevant (but disorder irrelevant) stimuli may also be disrupted in OCD [84], but the direction of this effect is not consistent [86]. A recent study, using disorder irrelevant conditioned stimuli, showed that like in post-traumatic stress disorder [87], OCD patient have deficits in the recall of extinction memories and show reduced activation in the medial portion of the OFC during extinction learning, but there were no group differences in amygdala activation [88]. These data, although somewhat inconsistent, indicate that the contribution of anxiety to habit biases in OCD cannot be ruled out, suggesting there is room for a more classic interpretation of the relationship among obsessions, anxiety and compulsions, that can coexist with a ‘COD’ account, which hypothesizes that there is a bidirectional mechanism of maladaptive symptom reinforcement. At a minimum, it is likely that trait anxiety may play a role in targeting OCD patients' general tendency towards excessive habit learning specifically to the avoidance domain, rather than towards ‘appetitive compulsivity’, e.g. stimulant drug addiction (but see [89] for a negative reinforcement hypothesis of addiction). This postulate awaits further study.

With this in mind, the considerable neurobiological, pharmacological and genetic heterogeneity of OCD might be explained by understanding how trait anxiety, as an independent contributor to compulsive avoidance habit learning, fits into a trans-diagnostic model of the disorders along the respective compulsive and anxiety spectrums. In other words, it may be the case that there are many routes to the OCD phenotype, and that dysfunction in habit learning and trait anxiety are independent, yet interacting diatheses. Future research will need to test this possibility, which is in the spirit of the recent National Institute of Mental Health's Research Domain Criteria initiative [90], a major goal of which is to develop therapeutic strategies that can target biologically defined traits that are presumed to be heterogeneous within diagnostic categories of the Diagnostic and Statistical Manual of Mental Disorders 5 [91]. Within this framework, it is plausible that trans-diagnostic dimensions, for example, habit learning, could be specifically targeted by certain treatments, for example, dopamine (D2) receptor antagonists (which are particularly effective in patients with co-morbid tics [92], or behaviour modification techniques, such as response prevention or habit reversal therapy. Likewise, trait anxiety might respond preferentially to selective serotonin reuptake inhibitors, through their anxiolytic properties [93], or exposure therapy, which is aimed at extinguishing inappropriate Pavlovian fear responses.

Whether there is a specific causal role for obsessions in this formulation is unclear. It is possible that obsessions in OCD reflect dysfunction in an entirely independent process that interacts with compulsivity in a bidirectional fashion, much like what we have proposed for anxiety. However, the importance of obsessions in OCD has recently been called into question with the observation that ‘Pure O’, that is patients who experience obsessions in the absence of compulsive behaviour, may be a clinical misnomer. Williams *et al*. [94] found that these patients appear to exhibit previously overlooked mental compulsions and compulsions to seek reassurance. Another interesting possibility is that the historical distinction between obsessions and compulsions in OCD may be superficial and that obsessions are a form of compulsive, automatic thought. In this way, both obsessions and compulsions could be considered products of a disrupted goal-directed system, leading to over-active automatic thoughts (obsessions) and actions (compulsions), rather than being discrete traits that interact with one another.

## 5. Summary and conclusion

The hypothesis that a shift from goal-directed to habitual control over action mediates compulsivity in OCD ties well with the neurobiological and pharmacological basis of habit learning in rodents and humans. This hypothesis also accords well with the neurocognitive profile of motor inhibition failures in OCD that are observed following repetition of action [95]. The ‘COD’ model of OCD proposed here has implications outside of the specific domain of this disorder. It is a plausible interpretation that the experience of premonitory ‘want’, or ‘urge’, reported in not only OCD but also substance-dependent individuals and tic disorders, may be a consequence of excessive stimulus–response associations. Future research should test this exciting possibility, articulated first by George Eliot in Silas Marner [96, p. 17]:

…repeating some trivial movement or sound, until the repetition has bred a want, which is incipient habit.
